# Design and Evaluation of Real-Time Data Storage and Signal Processing in a Long-Range Distributed Acoustic Sensing (DAS) Using Cloud-Based Services

**DOI:** 10.3390/s24185948

**Published:** 2024-09-13

**Authors:** Abdusomad Nur, Yonas Muanenda

**Affiliations:** 1 Addis Ababa Institute of Technology, Addis Ababa University, King George VI St, Addis Ababa 1000, Ethiopia; 2 Institute of Mechanical Intelligence, Scuola Superiore Sant’Anna, Via G. Moruzzi 1, 56124 Pisa, Italy

**Keywords:** distributed acoustic sensing, cloud computing, sensors, fiber, distributive sensing, remote sensing

## Abstract

In cloud-based Distributed Acoustic Sensing (DAS) sensor data management, we are confronted with two primary challenges. First, the development of efficient storage mechanisms capable of handling the enormous volume of data generated by these sensors poses a challenge. To solve this issue, we propose a method to address the issue of handling the large amount of data involved in DAS by designing and implementing a pipeline system to efficiently send the big data to DynamoDB in order to fully use the low latency of the DynamoDB data storage system for a benchmark DAS scheme for performing continuous monitoring over a 100 km range at a meter-scale spatial resolution. We employ the DynamoDB functionality of Amazon Web Services (AWS), which allows highly expandable storage capacity with latency of access of a few tens of milliseconds. The different stages of DAS data handling are performed in a pipeline, and the scheme is optimized for high overall throughput with reduced latency suitable for concurrent, real-time event extraction as well as the minimal storage of raw and intermediate data. In addition, the scalability of the DynamoDB-based data storage scheme is evaluated for linear and nonlinear variations of number of batches of access and a wide range of data sample sizes corresponding to sensing ranges of 1–110 km. The results show latencies of 40 ms per batch of access with low standard deviations of a few milliseconds, and latency per sample decreases for increasing the sample size, paving the way toward the development of scalable, cloud-based data storage services integrating additional post-processing for more precise feature extraction. The technique greatly simplifies DAS data handling in key application areas requiring continuous, large-scale measurement schemes. In addition, the processing of raw traces in a long-distance DAS for real-time monitoring requires the careful design of computational resources to guarantee requisite dynamic performance. Now, we will focus on the design of a system for the performance evaluation of cloud computing systems for diverse computations on DAS data. This system is aimed at unveiling valuable insights into performance metrics and operational efficiencies of computations on the data in the cloud, which will provide a deeper understanding of the system’s performance, identify potential bottlenecks, and suggest areas for improvement. To achieve this, we employ the CloudSim framework. The analysis reveals that the virtual machine (VM) performance decreases significantly the processing time with more capable VMs, influenced by Processing Elements (PEs) and Million Instructions Per Second (MIPS). The results also reflect that, although a larger number of computations is required as the fiber length increases, with the subsequent increase in processing time, the overall speed of computation is still suitable for continuous real-time monitoring. We also see that VMs with lower performance in terms of processing speed and number of CPUs have more inconsistent processing times compared to those with higher performance, while not incurring significantly higher prices. Additionally, the impact of VM parameters on computation time is explored, highlighting the importance of resource optimization in the DAS system design for efficient performance. The study also observes a notable trend in processing time, showing a significant decrease for every additional 50,000 columns processed as the length of the fiber increases. This finding underscores the efficiency gains achieved with larger computational loads, indicating improved system performance and capacity utilization as the DAS system processes more extensive datasets.

## 1. Introduction

Distributed Acoustic Sensing (DAS) is a technology that utilizes optical fibers to detect and measure acoustic signals along their entire length [1,2]. By sending laser pulses through the fiber and analyzing the backscattered light acoustic disturbances, DAS systems can convert fiber optic cables into continuous, high-resolution sensors [3,4]. This capability enables DAS to monitor vast areas or long infrastructure spans with a single fiber, making it ideal for applications such as oil and gas pipeline monitoring, the structural health monitoring of bridges and buildings, and environmental sensing. DAS systems are versatile, resilient to harsh environments, and can integrate seamlessly into existing fiber optic networks [5,6], offering a cost-effective solution for real-time acoustic monitoring, and improving operational efficiency across various industries.

### 1.1. DAS Sensing Principle

Distributed Acoustic Sensing (DAS) operates by detecting changes in the coherent Rayleigh back-scattering of a probe laser within an optical fiber [7]. This principle leverages several physical effects—such as the elasto-optical effect, thermo-optic effect, and thermal expansion—to measure the ambient physical field. When these effects modulate the optical characteristics of the probe laser (amplitude, phase, frequency, etc.), detecting and demodulating these changes allows the determination of the physical field along the fiber’s length.

**Figure 1:**
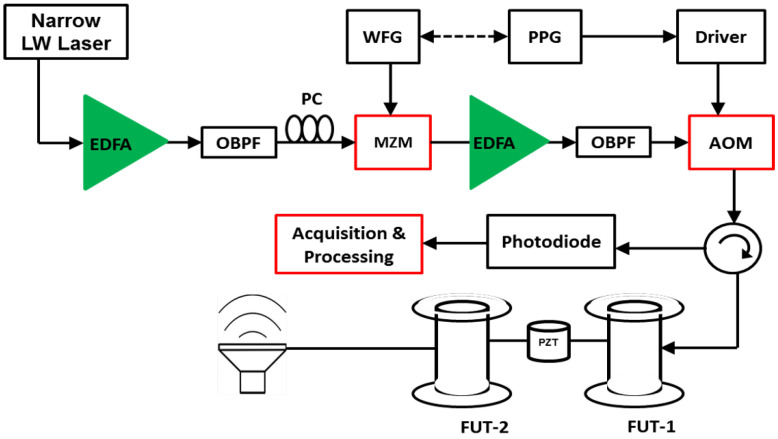
Experimental setup of a distributed vibration sensor using a ϕ-OTDR scheme in direct detection [8].

The most common implementation of a long-ranged DAS is shown in Figure 1. First, light from a narrow linewidth laser is amplified with an Erbium-Doped Fiber Amplifier (EDFA), whose amplified spontaneous emission is filtered using the Optical Bandpass Filter (OBPF). The amplified light then passes through an acousto-optic modulator (AOM), which generates the pulses which are subsequently amplified and filtered using a second pair of EDFA and OBPF. The coherent Rayleigh scattering signal carrying information about the fiber’s condition is directed back through the return port of the circulator to a photodetector which converts it to an electrical signal. Then, a data acquisition system is used to acquire the traces in real-time for subsequent processing to extract changes in physical parameters such vibration, strain and temperature along the fiber.

In ϕ-OTDR, the propagation phase of the probe pulse is influenced by vibration [9]. The longitudinal strain of the fiber caused by external vibration is denoted as ϵ, and the resulting change in the fiber’s effective refractive index $\Delta n_{\mathrm{eff}}$ is given by [10,11]:

(1) $$\Delta n_{\mathrm{eff}}=\gamma n_{0}\epsilon$$

where γ is the elasto-optical coefficient, and $n_{0}$ is the original refractive index of the fiber. Simultaneously, the length *l* of the fiber segment under vibration also changes, expressed as Δl=ϵ·l. The additional phase induced by this vibration is [10,11]:

(2) $$\Delta \phi =\left(1+\gamma \right)n_{0}kl\epsilon$$

where $k=\frac{2\pi }{\lambda }$ is the wavenumber of the laser light (with λ being the wavelength).

Due to the finite width of the probe pulse, backscattering from different points along the fiber overlaps, causing internal pulse interference. This interference pattern varies with changes in the additional laser phase and the external vibration. By detecting this pattern, vibration can be qualitatively identified. Furthermore, since vibration is linearly proportional to the phase change in backcattering signal indicated by the equation above, it can be quantitatively measured using phase demodulation. This process forms the core sensing principle of DAS with ϕ-OTDR [12].

### 1.2. Measurements in a Distributed Acoustic Sensing

For a DAS based on ϕ-OTDR, a number of traces are acquired [13], and the typical processing to identify vibration location and frequency involves computing the differential intensity or phase changes for samples corresponding to each point along the fiber, which is a matrix subtraction operation.

More precisely, quantitative measurements of the vibration include the demodulation of the phase variation in subsequent periods for each spatial location, using algorithms which have large time complexities, which at times impact the dynamic performance when the sensing distance is long. In this work, we provide the design of a cloud computing system for data storage and signal processing by focusing on matrix subtraction and FFT, the most commonly used operations involved in post-processing in distributed sensing.

The schematic and early configurations of time domain DOFSs based on coherent Rayleigh back-scattering are similar to those of simple OTDR. However, the sources used in such sensors are stabilized narrow linewidth lasers, and the phenomenon exploited is coherent Rayleigh back-scattering. The areas of applications and the types of measurements differ, and hence, the events which can be detected are also different. Typical applications of such a system include intrusion detection in perimeter security [14] and in long-distance oil and pipeline monitoring [15].

**Figure 2:**
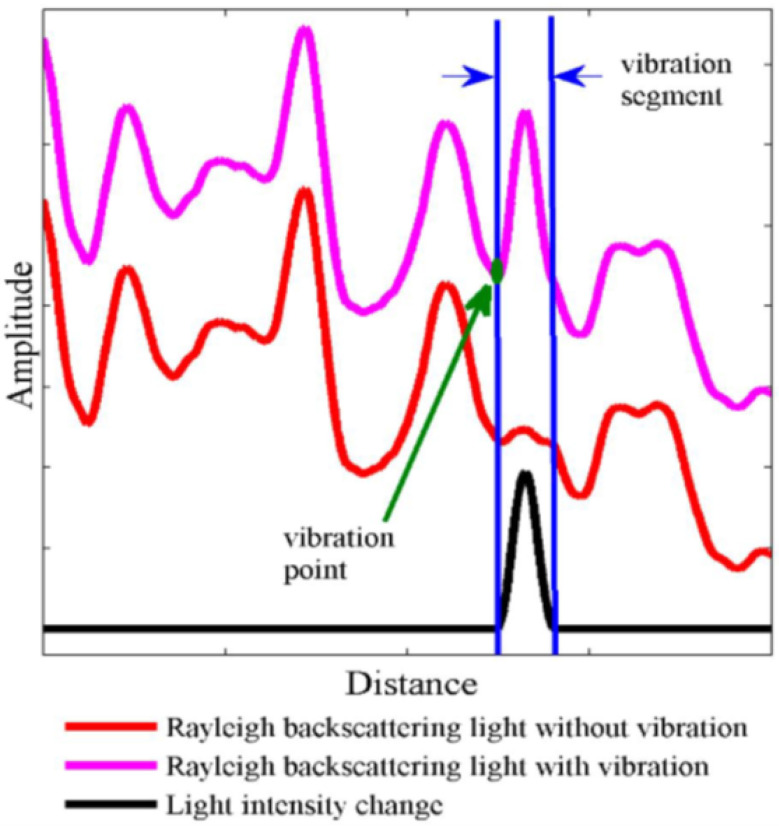
Intrusion detection using a ϕ-OTDR sensor [16].

The measurement principle used in ϕ-OTDR is qualitatively described in Figure 2 [14]. As shown, in the presence of an external agent such as a vibration source or an intrusion, a phase change will be induced in the back-scattered signal since touching the fiber changes the refractive index and the optical path length of the light. The change in phase will be reflected in the measured intensity of the back-scattered Rayleigh trace. If two independent measurements $X_{i}$ and $X_{j}$ are taken at two different time instances i and j, the traces will show a difference in the intensity level. As shown in the diagram, this can be rendered visible by computing a differential trace $\Delta X=X_{j}-X_{i}$, which is the arithmetic difference of the amplitude levels per distance along the fiber.

In a DAS based on ϕ-OTDR, the acquisition of a number of independent traces can be used to determine local vibrations over long distances at the granularity of the spatial resolution. The frequency of sampling for a given location is dependent on the fiber length L and the group velocity of the light, as determined by its speed in free space c, and the group refractive index n. The total measurement time $T_{meas}$ for a set of traces is the product of the round trip time of light in the fiber $T_{R}$ and the number of averages per acquired trace $N_{av}$. The achievable sampling rate is then given by:

(3) $$f_{s}=\frac{1}{T_{meas}}=\frac{1}{N_{av}T_{R}}=\frac{1}{N_{av}2n\frac{L}{c}}=\frac{c}{2N_{av}nL}$$

and according to Nyquist’s Sampling Theorem, the maximum frequency of vibration that could be reconstructed is:

(4) $$f_{v-max}=\frac{1}{2}f_{s}=\frac{1}{4}\frac{c}{LnN_{tr}}$$

Equation (4) shows that the ϕ-OTDR measurement of high-frequency vibrations at long distance can only be performed if the number of averages can be reduced. In the ideal case of $N_{av}=1$, using a single-mode fiber of a few kilometers and a coherent optical source at 1550 nm, the detectable frequency of vibration is in the order of a few tens of kHz. This is the reason behind the use of ϕ-OTDR in long-range vibration-monitoring applications. However, it is worth mentioning that the spatial resolution performance of OTDR sensors for fast online measurements is, in general, lower compared to other forms of sensors using Rayleigh back-scattering based on Optical Frequency Domain Reflectometry (OFDR) [16].

Even though the main application of DAS based on coherent/phase-sensitive OTDR is in intrusion detection and vibration frequency measurements, it has also been used to measure temperature and strain [17,18]. The main principle for strain and temperature measurement consists of the fact that either temperature or train variations will result in a change in the local phase.

From the expression of the field for coherent Rayleigh back-scattering given in the equation [16], we clearly see that the phase of the back-scattering is dependent on the refractive index of the fiber, the distance in which the phase change occurs, and the wavelength of the incident light [19].

The multiplicity of data in distributed sensing has created the necessity to store and process data on a large scale. This is specifically true for real-time applications requiring the monitoring of dynamic events with latencies much faster than those can be obtained by simple processing systems. While cloud storage and computing have been used for data processing in a number of online services, their use in long-range distribution sensing has not been investigated properly.

One key step forward in this direction requires quantifying the type, number, and specifications of resources required for a scenario of real-time monitoring with dynamic distributed sensing. Specifically, the intermediate data handling and signal processing in a DAS system involve the acquisition of multiple traces and subsequent processing for denoising, spectral computations, and phase demodulation techniques [20]. Given that, often, knowing the exact storage and processing resources for a given system requires tests on real systems which are costly, it is convenient to use tools which simulate the cloud architecture, and allow the prediction of the expected performance results in simple, readily available machines.

One such tool is CloudSim, which has been used in many design scenarios spanning a number of applications. The tool has been widely used by researchers and engineers with various approaches including ones for simple processing [21], adaptations for the simulation of distributed functions as a service (SaaS) [22].

The various scheduling algorithms in cloud environments have been captured by the simulation tool and shown to serve as effective design tools to determine cloud solutions for multiple scenarios. Among others, CloudSim has been used to simulate computations in smart grid by studying parameters such as the number and bandwidth of virtual machines as well as the RAM and cloudlet length [23]. Specifically, the time share allocation policy has been shown to offer a greater execution time for a higher number of cloudlets compared to the space-sharing allocation policy, and the same is true when the VM bandwidth and RAM have been increased.

More recently, the increase in the number and complexity of devices in the IoT architecture, which necessitated edge and cloud computing, has also inspired the dedicated development of a cost-effective simulation tool aimed at capturing the distribution of the load of computation by optimizing the device specifications and power consumption [24]. This and other scalable simulators enable the analysis of multiple parameters for a large number of devices and offers application interfaces for visualization [25], including in the placement and optimization of edge server computing [26].

While different aspects of DAS have been studied in detail, there are limited investigations of the tools and approaches for rendering long-range distributed sensors suitable for real-time monitoring. This is, to the best of our knowledge, the first study of the cloud simulation tool for modeling the signal processing in a sample distributed fiber-sensing system.

### 1.3. Challenges of DAS Long-Range Sensing

Distributed dynamic fiber optic sensors have interesting applications in many safety and integrity monitoring systems. Among others, DAS, which involves the use of coherent Rayleigh back-scattering [27] signals whose local phase and intensity are sensitive to vibrations and temperature, has attracted the interest of a wide array of research and development [28,29], focused mostly on improving the cost effectiveness and precision [30,31] of the interrogation scheme. However, the continuous, real-time monitoring of environmental parameters in a DAS require the efficient, scalable, and reliable storage and processing of a large amount of data. For continuous monitoring in long sensing ranges, the problem of managing data generated by a DAS system enters in the domain of “5Vs” comprising big volume, high velocity and veracity, and a large variety [32] as well as variability, hence the critical need for big data storage and analytics tools including those leveraging the cloud. A recent survey shows that tools in data science and machine learning are projected to have a strong impact on the next generation of distributed optical fiber sensors used in environmental monitoring, including in smart cities and Internet of Things (IOT) [33]. So far, owing to the large number of sensors generating heterogeneous and multi-dimensional data, Big Data storage services have been used to manage data from networks of electronic sensors for smart environmental monitoring [34] including those of fiber seismic sensor networks [35]. Since they have a capacity to aggregate information from a number of sensors, cloud-based IoT applications have significant impact on the advance in techniques used in smart cities [36]. Hence, long-range, single and multi-parameter distributed sensing systems will benefit from the inclusion of well-developed tools in cloud storage and processing services.

With respect to data storage and handling techniques, traditional, so-called Sequential Query Language (SQL) databases use simple relational databases which are not easy to adapt when dealing with complex and dynamic datasets. More recent schemes use NoSQL (Not only SQL) databases [37], which are optimized for storing data with various shapes and sizes and exhibit improved flexibility to store a large amount of multi-dimensional and unstructured information. In addition to such flexibility and dynamic behavior, NoSQL databases have significantly lower latency of access. Although a number of cloud storage services exist, including Google Drive, Dropbox, One Drive, Media Fire, etc., Amazon’s DynamoDB offers unique features, as it is a server-less, NoSQL database which offers low latency even at huge data volumes [38]. These features have enabled its wide use in the development of scalable tools in, among others, the shopping, banking, transportation and entertainment industries, where consistently fast responses per access are observed even in peak load conditions involving tens of millions of requests. Despite the proven promises of such big data schemes, their use in distributed fiber optic sensing has not been closely studied [33]. In this work, we assess and report on the feasibility and scalability of using DynamoDB for sample DAS data handling.

In a continuous monitoring scenario using any distributed sensing scheme, multiple traces are acquired at rates whose upper limits are determined by the fiber round trip time $t_{R}$. Each individual trace contains information on the sensing parameter for all locations along the fiber. When the time evolution of each parameter is observed, it provides information. For instance, in a typical DAS scheme used in acoustic vibration measurements, the maximum measurable frequency $f_{v-max}$ for a sensing distance *S* and number of averages $N_{tr}$ is given by:

(5) $$f_{v-max}=\frac{1}{4}\frac{\frac{c}{n_{g}}}{SN_{tr}}$$

where $n_{g}$ is the group refractive index, and c is the speed of light in free space. For a sensing range of S=1 km, in which $N_{tr}=1$ (with no averaging), the maximum measurable frequency of vibration is 50 kHz. Considering that typically tens of averages are made to enhance the SNR, the effective measurable frequency can be few as hundreds of Hz. For a 100 km sensing range and a minimal number of averages, the measurable vibration frequencies are in the tens of Hz range, which corresponds to tens of milliseconds. While raw traces need to be taken at rates of the RTT of the fiber, the storage of intermediate data obtained from them and subsequent processing needs to scale and maintain its efficiency with the duration of acquisition of the number of samples acquired per acquisition.

In the realm of cloud-based sensor data management, the journey expands beyond mere storage mechanisms into the dynamic landscape of computational analysis. Our focus now shifts to the design and evaluation of a robust system tailored for diverse computations on these data, aimed at unveiling insights into the performance metrics and operational efficiencies. Fueling this exploration is the CloudSim framework, a versatile simulation toolkit renowned for its ability to model complex cloud computing infrastructures and services. Within this framework, our system takes shape, comprising essential elements such as a resilient data center, agile cloudlets representing computational tasks, virtual machines (VMs) configured with varying specifications, and a strategic resource broker orchestrating resource allocation. At the core of our computational powerhouse lies a host with formidable capabilities with different specs including MIPS of processing power, RAM, storage, and a network bandwidth which will be used by our VMs. These foundational attributes lay the groundwork for simulating intricate cloud infrastructures and gauging performance benchmarks effectively.

Through a series of simulations, we delve into two distinct scenarios, each illuminating the efficiency and adaptability of cloud computing in handling data acquisition systems tasks. These simulations involve batch-processing operations, leveraging differential operation techniques and Fast Fourier Transform (FFT) computations on input data, all orchestrated within the CloudSim environment. The goal is not just to showcase the computational capabilities but also to unravel insights into cost effectiveness and performance metrics in cloud-based computational scenarios.

### 1.4. DynamoDB

Amazon DynamoDB is a fully managed NoSQL database service that offers seamless scalability along with fast and predictable performance [39]. The key features of DynamoDB are encryption at rest, which removes the complexity and administrative burden of protecting sensitive data and lets you offload the administrative burdens associated with running and scaling a distributed database, such as hardware provisioning, setup and configuration, replication, software patching, and cluster scaling [40]. Tens of thousands of micro-services make up DynamoDB. The metadata service, request routing service, storage nodes, and auto-admin service are a few of the primary functions of DynamoDB as it is described in [38]. A high-level overview of the architecture of the Amazon platform, wherein page rendering components generate dynamic web content by referencing numerous other services is shown in [41].

### 1.5. Cloud Computing and CloudSim

The term “cloud computing” refers to a computer paradigm that uses shared computing resources to process applications instead of relying on local servers or personal devices. Grid computing, which uses the underutilized processing power of every computer connected to a network to address problems too complex for any single standalone system, is comparable to cloud computing [42,43]. Cloud computing is becoming more preferable for a number of reasons. Cloud services are flexible, have dynamic behavior, are server-less, and NoSQL databases have significantly lower latency of access. Some of the cloud storage services that exist include Google Drive, Dropbox, One Drive, Media Fire, etc. These features have enabled its wide use in the development of scalable tools in, among others, the shopping, banking, transportation, and entertainment industries, where consistently fast responses per access are observed even in peak load conditions involving tens of millions of requests [44].

Cloud system elements including data centers, virtual machines (VMs), resource provisioning policies, and the full cloud system and behavior could be modeled with the help of CloudSim tools. The application provisioning approaches that it employs are generic and easily extensible. They can currently simulate and model cloud computing environments made up of single clouds as well as clouds that are interconnected (cloud federation). It also provides access to customized APIs for provisioning methods and policy implementation for virtual machine (VM) allocation in scenarios involving interconnected clouds. In their research on cloud resource provisioning and energy-efficient data center resource management, a number of researchers from companies like HP Labs in the United States are utilizing CloudSim [45]. The CloudSim simulator was mostly created in Java and is freely accessible to the public under an LGPL license. Detailed discussions on cloud computing architecture can be found in [46,47,48].

## 2. Schematic of System for Sending DAS Data to AWS DynamoDB and Signal Processing with CloudSim

**Figure 3:**
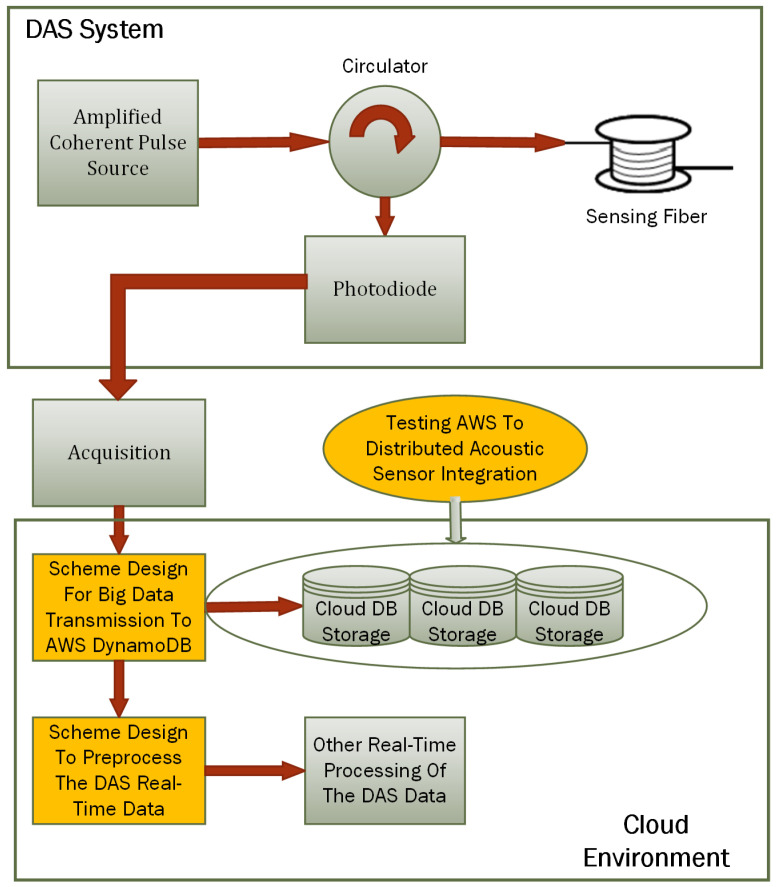
Block diagram of the developed system.

Figure 3 shows a system designed to integrate Distributed Acoustic Sensing (DAS) with AWS cloud services, specifically, AWS DynamoDB.

In the cloud environment, the first major component is the scheme designed for big data transmission to AWS DynamoDB. This involves creating and implementing a system to efficiently transmit the large volumes of data acquired by the DAS system to AWS DynamoDB for storage. The cloud DB storage, represented by distributed storage solutions provided by AWS DynamoDB, stores the transmitted DAS data. Another crucial component is the scheme designed to preprocess the DAS real-time data, which refer to the preprocessing steps necessary to prepare the DAS data for storage and further analysis. A critical aspect of this system is the integration testing labeled as “Testing AWS to Distributed Acoustic Sensor Integration”. This step involves validating the integration between the DAS system and AWS cloud services.

### 2.1. Scheme Design for Big Data Transmission to AWS DynamoDB: Testing AWS to Distributed Acoustic Sensor Integration

**Figure 4:**
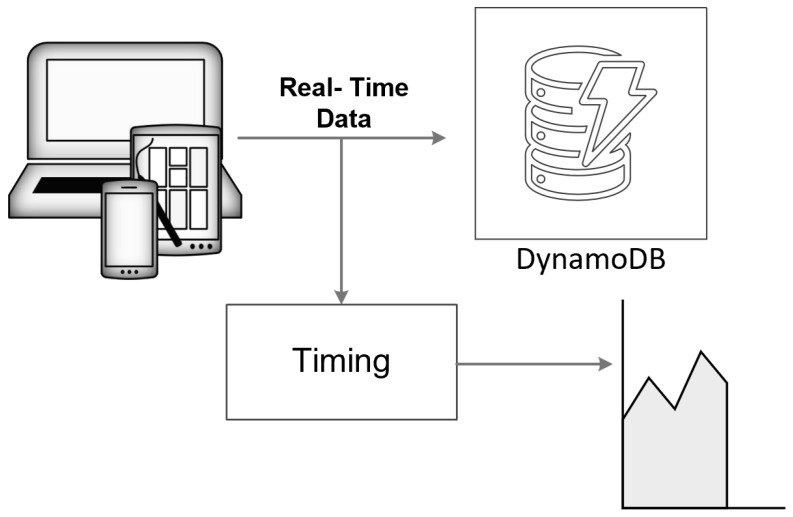
Schematic representation of the connection of the DAS sensor system to DynamoDB.

Figure 4 shows the schematic representation of the scheme to test the integration of AWS DynamoDB with DAS. Creating an AWS account with DynamoDB as one of its services is the first step in leveraging the powerful cloud-based database solution provided by Amazon Web Services. After setting up the account, the next task is to create a table within DynamoDB, which can be accomplished using either the DynamoDB console or programmatically through Python. The Python approach typically involves using the Boto3 library, which allows for seamless interaction with AWS services. Once the table is created, it becomes the repository for the incoming data. In this case, the data comprise trace samples, which are sent to the DynamoDB table. The process of sending data involves capturing the start time and end time to accurately record the duration required for each data transmission. This measurement is crucial for understanding the performance and efficiency of data handling within the database.

To further analyze the performance, the process is repeated in a loop, varying the sample size to simulate data received from fiber sensors over different distances, ranging from 1 km to 110 km. Each iteration in the loop involves sending a different volume of data to the DynamoDB table and recording the corresponding time taken for the transmission. This detailed and repetitive testing helps in understanding how the data size impacts the transmission time and provides insights into the scalability and efficiency of the database service when handling large datasets. Through this comprehensive approach, we can gather valuable metrics on the operational performance of DynamoDB involving large-scale data collection from fiber optic sensors.

### 2.2. Design of a Signal-Processing Scheme in a Distributed Acoustic Sensor Using CloudSim

As described in previous section, CloudSim is used to simulate different scenarios of cloud-based systems in order to understand and test the different working mechanisms and services of the cloud infrastructure.

**Figure 5:**
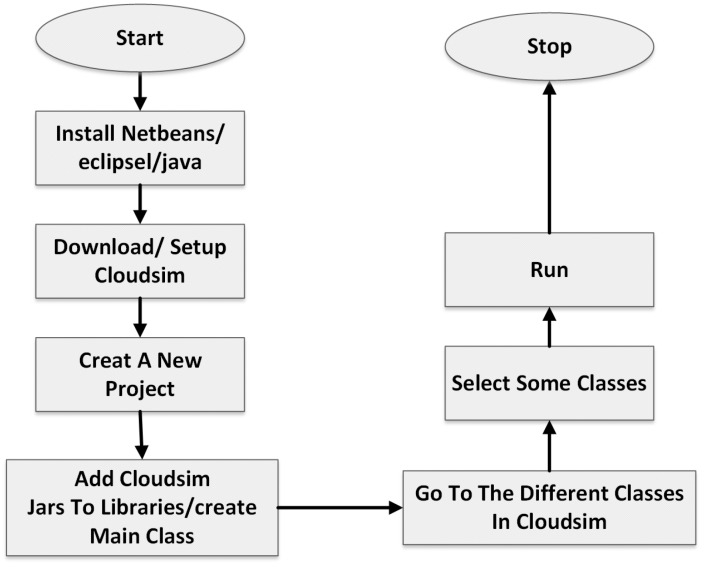
Steps to use CloudSim.

Figure 5 provides a step-by-step guide to setting up a cloud simulation project using CloudSim (cloudsim-5.0), from the installation of necessary software to the execution of the simulation. The process begins with the installation of the chosen IDE or Java, providing the necessary tools for the development and execution of the CloudSim project. Once the IDE or Java is installed, the next step involves downloading and setting up CloudSim, a framework for the modeling and simulation of the cloud computing infrastructures and services. After CloudSim is set up, a new project is created within the IDE. This project will serve as the workspace for developing the cloud simulation. To facilitate the simulation, CloudSim JAR files are added to the project libraries, and a main class is created. The main class serves as the entry point for the execution of the project. In parallel to adding CloudSim JARs to the libraries, understanding the functionalities provided by these classes and how they can be utilized in the simulation is necessary. Once the project setup is complete, some classes are selected, presumably for customization or to define the specifics of the simulation. With the project set up and configured, the simulation can be run. The process concludes with the termination of the simulation, indicated by the “Stop” node in the flowchart.

**Figure 6:**
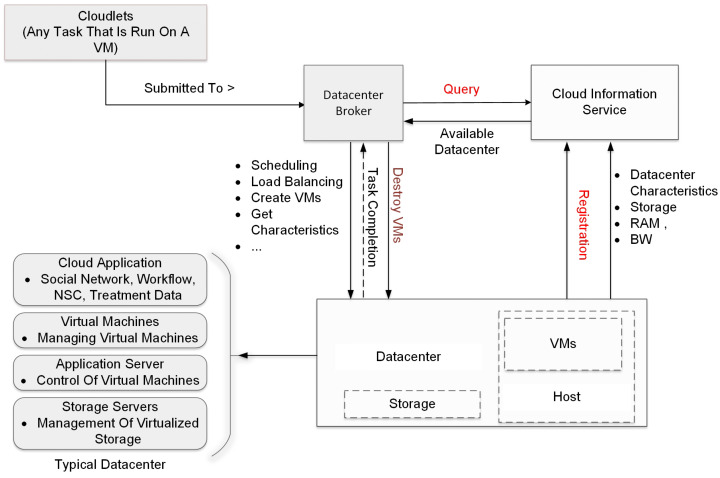
Block diagram of simulation flow for the basic scenario.

The simulation flow for the basic scenario process in the CloudSim framework is shown in Figure 6. The simulation process begins with the initialization of the CloudSim environment. This is the first step in setting up the simulation and involves preparing the necessary resources and parameters for the simulation to run. Following the initialization, a data center is created. The data center is a crucial component of the cloud infrastructure, housing the physical resources such as servers, storage devices, and networking equipment. It is responsible for managing the execution of cloudlets, which are tasks that run on virtual machines (VMs). The virtual machines are needed to simulate nodes in an actual cloud computing infrastructure so as to evaluate the performance needed for processing large datasets generated during continuous, real-time monitoring in a long-range DAS, which would be challenging to perform with ordinary processing systems. Once the data center is set up, a data center broker is established. The broker acts as an intermediary between users and the data center, managing the distribution of tasks and the allocation of resources. It is responsible for scheduling tasks, balancing the load among available resources, creating VMs, and determining the fee characteristics for the use of resources. After the broker is set up, the next step involves the creation of VMs and cloudlets. VMs are virtual representations of physical machines, providing an environment for tasks to run. Cloudlets, on the other hand, represent the tasks that need to be executed. These VMs and cloudlets are added to their respective lists for management purposes. The lists of VMs and cloudlets are then submitted to the broker. The broker takes these lists and schedules the tasks on the available VMs based on various factors such as resource availability, task requirements, and scheduling policies. With everything set up, the simulation can now start. The tasks are executed on the VMs, and the simulation runs until all tasks are completed. Once all tasks are completed, the simulation is stopped. This marks the end of the simulation process. Finally, the results of the simulation are printed. This includes information such as the execution time of tasks, and the utilization of resources. These results provide valuable insights into the performance and efficiency of the cloud infrastructure. This sequence of steps provides a structured approach to simulating cloud environments, ensuring that all necessary components are properly set up and managed, and that the simulation results are accurately recorded and presented. The arrows in the flowchart indicate the flow of tasks and information, showing how each component interacts with others in the simulation process. This helps in understanding the complex interactions and dependencies in a cloud environment.

**Figure 7:**
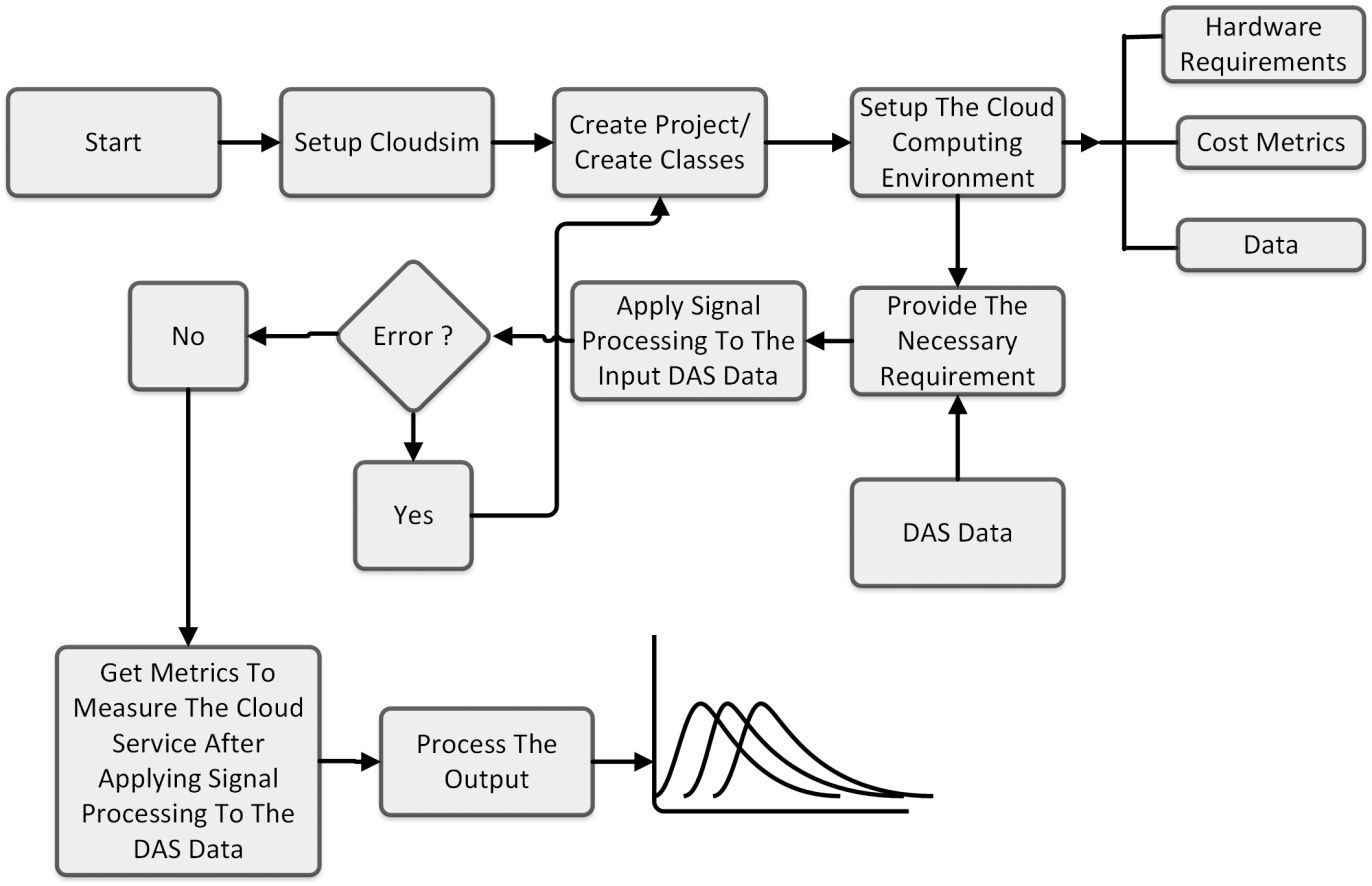
Schematic representation of the implementation of processing of DAS data in CloudSim.

The schematic in Figure 7 represents the implementation of the signal-processing system in a DAS sensor system using CloudSim. The process begins with the installation and setup of the CloudSim system. This involves downloading the necessary software, configuring the environment, and ensuring that all dependencies are correctly installed. This step lays the foundation for the rest of the process and is crucial for the successful execution of the simulation. Once CloudSim is set up, the next step is to understand how the system works. This involves studying the documentation, exploring the codebase, and familiarizing oneself with the various components and functionalities of CloudSim. This understanding allows for effective utilization of the system in the subsequent steps. After gaining a thorough understanding of CloudSim, the system requirements are defined. These requirements could include the hardware specifications, and the data that will be used in the simulation. These requirements are crucial, as they determine the resources that will be allocated for the simulation and the performance of the system. With the system requirements defined, the next step is to create a new project and classes within the system that meet the sensor system requirements. These classes could represent various components of the cloud infrastructure such as data centers, virtual machines, and cloudlets. Once the project and classes are set up, the simulation is started. The simulation runs twice using two different Java classes. One class is responsible for processing differential computation, while the other handles Fast Fourier Transform (FFT) computation. These computations are integral to the simulation, as they process the data and generate the results. During the simulation, if an error occurs, the process loops back to the step of creating classes. This allows for the correction of errors and ensures the smooth running of the simulation. If there are no errors and the system is functioning as expected, the simulation is stopped. Subsequently the outputs are produced. These outputs could include various metrics and results from the simulation. Finally, these outputs are processed in MATLAB for further analysis. This involves visualizing the results, performing statistical analysis, and using the results to make informed decisions about the cloud infrastructure.

## 3. Results and Discussions

To test the effectiveness of the proposed technique, a local Amazon DynamoDB storage using Python with the Boto3 interfacing feature is implemented. The scheme is tested with randomly generated intermediate data representing the samples of a generic long-distance monitoring with meter-scale spatial resolution for Distributed Acoustic Sensing (DAS). For a benchmark sensing along a 100 km distance with 5 measurements samples in each meter, a total of 500,000 samples of raw data are stored per trace. We then test the times needed for database access for the writing of the large volumes of data with respect to the benchmark, considering scalability in terms of various measurement durations with multiple traces and different sensing distance values in the range 200 m–100 km.

After we successfully design an efficient storage mechanism for the sensor data in the cloud environment, we will embark on a comprehensive journey to design and analyze a robust system for performing a variety of computations on sensor data in the cloud.

The first phase of our work involved the design of the computational system. We aimed to create a system that was not only capable of handling the vast amounts of sensor data but was also efficient in processing it. The system was designed with scalability in mind, ensuring that it could handle increasing volumes of data with minimal impact on performance. Once the system was designed, we moved on to the performing of computations on the DAS sensor data. The primary objective here was event extraction by applying different computations like differential and FFT operations on the DAS data to extract events from these datasets. The final phase of our work was the performance measurement of the system. This involved a series of tests, where we varied different parameters of the cloud computing system. These parameters included factors like the length of the sensing fiber, the complexity of the computations, the number of concurrent users, and the network bandwidth, among others. By varying these parameters, we were able to gauge the system’s performance under different conditions. The results of these tests provided valuable insights into the cloud system’s robustness, scalability, and efficiency in using it in the DAS sensor system.

### 3.1. Evaluation of Storage Times for DAS Data Using Amazon DynamoDB

**Figure 8:**
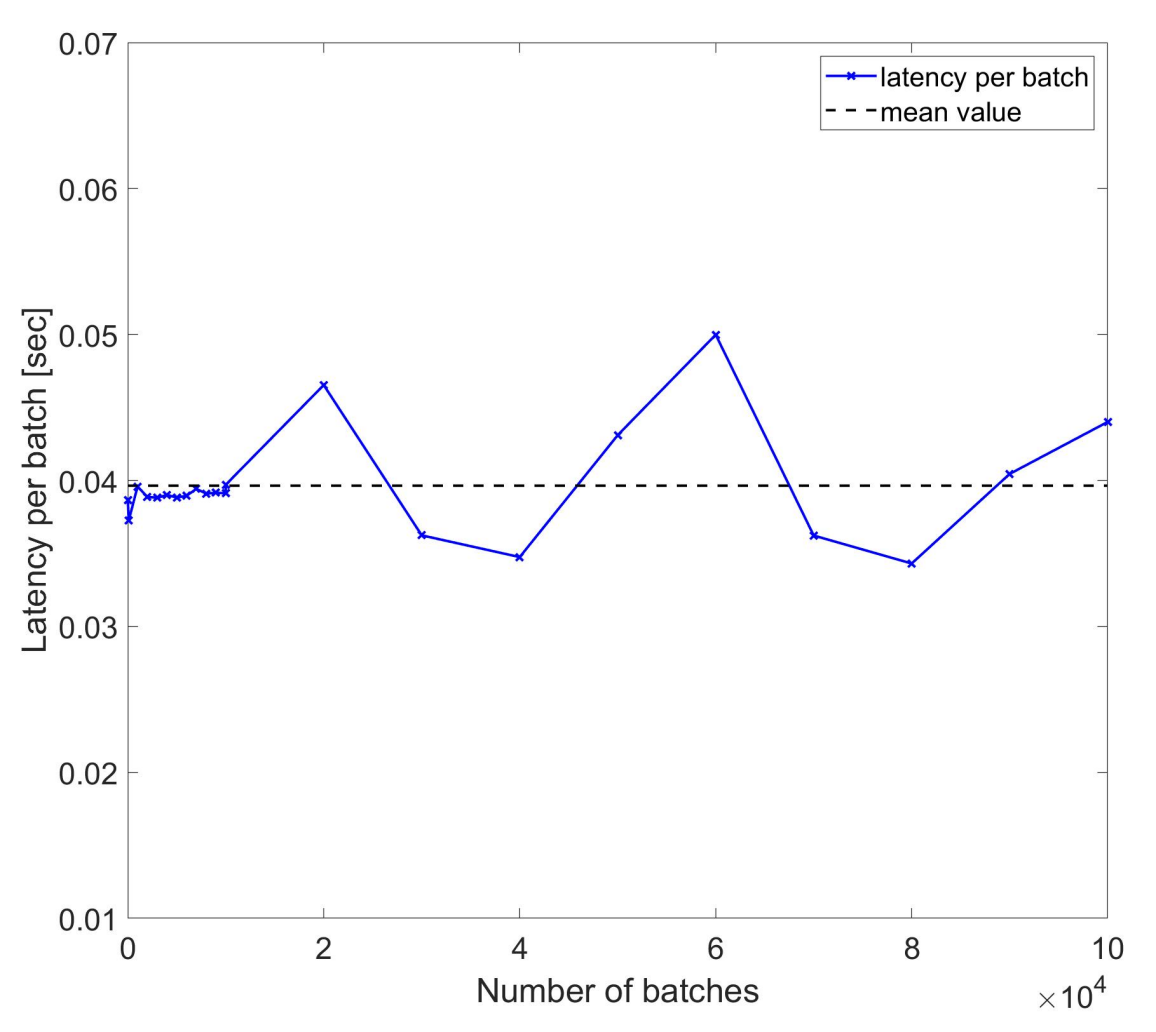
Latency per batch of DynamoDB access for sample number of batches used to write trace samples.

The first set of performance evaluations consisted in the measurement of the latency for varying durations (varying trace counts) for multiple counts of the 500,000 samples, with each sample saved in a double-precision floating point format having a size of 8 bytes. Since the maximum data size for an Amazon DynamoDB input is 400 kB, data were stored using batches of 50,000 samples each. For a 100 km sensing distance, there will be 10 batches for storing samples in the whole trace. Considering an RTT of 1 ms for the benchmark sensing range, the number of batches for measurement duration Tm will be given by Tm x10/0.001. Figure 8 shows the latency of storage for 20 different durations from 1 ms up to a few seconds. The measured latencies per batch have a standard deviation of ∼0.0036 s and a mean value of ∼0.0396 s, consistent with specifications of Amazon DynamoDB latencies for corresponding data sizes. Note that the time tags are dependent on the rate of acquisition of data, and in our simulation, we considered cases with rates of a few 100 s MS/s corresponding to nanosecond-scale sampling times.

**Figure 9:**
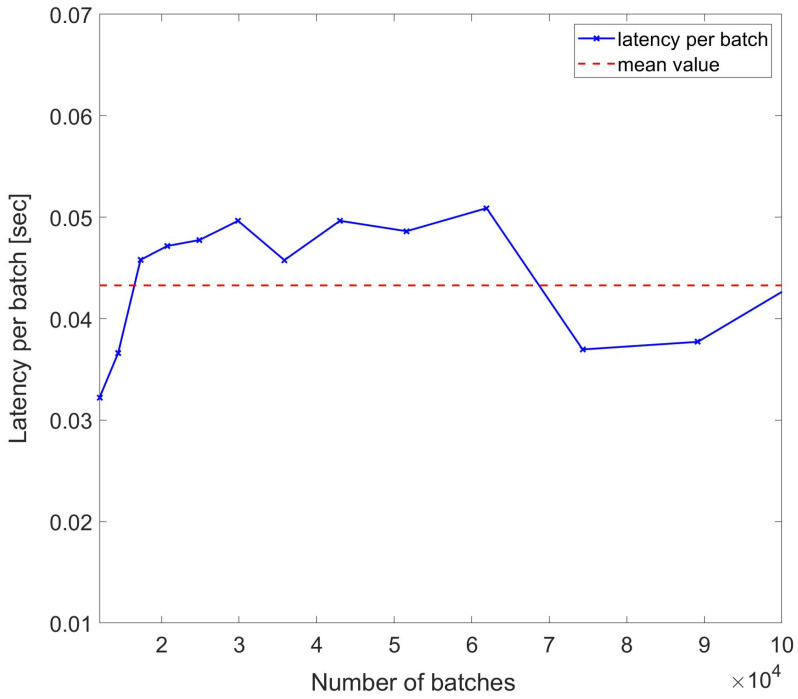
Latency per batch of DynamoDB access used to write trace samples with number of batches scaling with $2^{n}$ for each index n.

To assess the resilience of latencies per sample in wider sets of data, another set of measurements were performed, while the traces acquisition duration was varied nonlinearly with multiples of $2^{n}$. The results are reported in Figure 9, which confirms the resilience of the DynamoDB access against nonlinear variations in the batch length with the mean value being ∼0.0433 s. As shown, even if the batch length scales with $2^{n}$, the per-batch latency remains the same, and some larger datasets even have lower par-batch latency.

**Figure 10:**
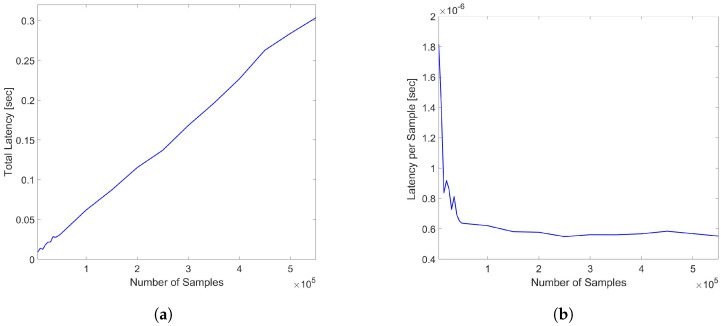
(**a**) Total latency of DynamoDB access (**b**) Latency per sample for varying trace sample sizes in the range of 5000–550,000 samples corresponding to 1–110 km sensing distances.

The evaluations of the total latency and the latency per sample for varying sample sizes corresponding to different sensing distances were also evaluated in the range from 5000 to 550,000 samples with the end points corresponding to sending distances of 1 km (5 samples per meter) and 110 km, respectively, and the results are reported in Figure 10a,b.

Note that if the probing pulses have narrow-enough width values and pulse peak power corresponding to a threshold of SNR in the sensing range, the higher number of samples also corresponds to the increasing spatial points and, hence, more resolved measurements with shorter pulses. As shown, while the total latency increases with the number of samples as expected, the latency per sample shows a decrease up to the cutoff at 50,000 samples and consistently stabilizes to a mean value of 5.6649 × $10^{-7}$ s, once again demonstrating the suitability of the DynamoDB service to a wide range of monitoring ranges or spatial resolution values. The fact that latency per sample has limited values regardless of sample size confirms the resilience of Amazon DynamoDB to increasing data sizes.

### 3.2. Evaluation of Computation Times of Signal Processing in a DAS Using CloudSim

The system is designed using the CloudSim framework. In our computational model, cloudlet lengths are rigorously calculated in terms of MI (Million Instructions) to precisely gauge the computational intensity of different operations. For instance, consider cloudlet0, where we perform the operation of subtracting the first row from all 1000 rows, each containing 50,000 columns. This operation entails 50,000 subtractions per row, resulting in a total of 50,000,000 subtractions across all rows. Assuming each subtraction necessitates 10 computations, the total computational load amounts to 500,000,000 computations or equivalently 500 MI. Subsequently, as we progress to cloudlet1 with an expanded column limitation of 100,000 columns, the computational complexity escalates twofold, yielding 1000 MIPS, denoting a doubling of the workload compared to cloudlet0. This methodical approach extends to cloudlet2, cloudlet3, and beyond, where each cloudlet’s MIPS is intricately derived as a multiple of its predecessor, portraying the systematic escalation in computational demands across the continuum of tasks.

The VM configurations exhibit significant diversity, with MIPS with different ranges, varying RAM, Processing Elements (PEs) spanning within different ranges, huge storage capacities, and bandwidth capacities in bits per second. The lengths of the cloudlets, which stand for computing jobs, range from 500 to 5500 MI; the file and output sizes from 500 MB to 5500 MB; and the PEs from 1 to 11. The system uses an AWS-style pricing model to determine prices for DynamoDB operations (read-and-write capacity units), EC2 instance utilization (MIPS, PEs, RAM, and storage), and storage fees per gigabyte per month. By means of this simulation, the system offers significant insights into the proper handling of the huge data that come from DAS sending.

We perform two operations using the CloudSim simulations in order to see the performance of cloud computing for DAS systems. The two scenarios are described as follows:

The CloudSim simulation is focused around batch-processing operations using differential operation and Fast Fourier Transform (FFT) on input data. The simulation creates a set of virtual machines (VMs) with varying configurations, including different amounts of RAM, MIPS, and storage capacity. The differential operation is performed by subtracting the first row from all rows, and to execute FFT calculations, it uses the JTransforms library of Java’s concept of applying FFTs. These VMs are deployed in a data center environment with one physical host provisioned with RAM, processing power, and storage that can support the VMs we will use in the simulation. The goal is to showcase batch-processing capabilities within a cloud computing context, demonstrating how VMs can efficiently handle different computations on large datasets. Additionally, the simulation calculates the processing times and accumulates the total processing time for each VM, emphasizing the practical aspects of cloud-based batch processing with different operations. We will provide different plots in order to analyze different aspects of cloud infrastructures. The plots will be provided in logarithmic scale, except some which will be specified.

**Figure 11:**
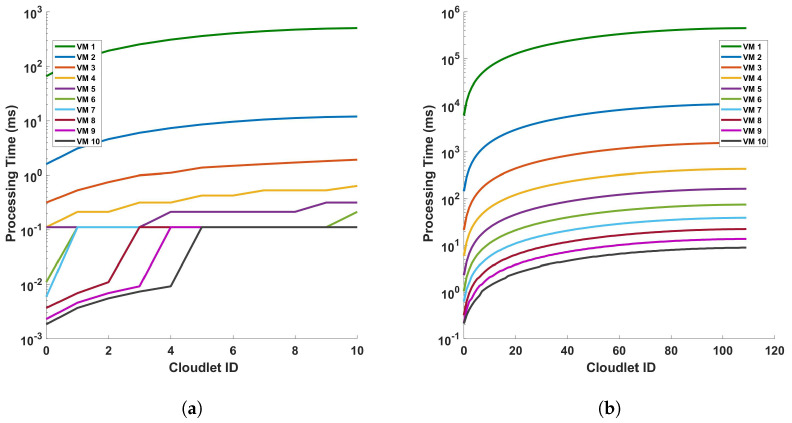
Analysis of processing time and cloudlet utilization for differential operations in DAS sensing system: a study on single cycle versus multiple cycles. The study focuses on two distinct scenarios: (**a**) a single cycle of measurement, and (**b**) a series of 10 consecutive cycles of measurement. The measurements are conducted in a 110 km long optical sensing fiber. Note that the number of cloudlets increases for each cloudlet ID in the horizontal axis.

Figure 11 provides an analysis of the processing time in relation to the length of cloudlets for differential operations. This analysis is conducted under two conditions: (a) a single cycle of measurement, and (b) a series of 10 consecutive cycles of measurement. The measurements are performed within a fiber optic cable that spans a distance of 110 km. The term ‘cloudlets’ in this context refers to the number of columns computed, which is directly proportional to the length of the fiber used for Distributed Acoustic Sensing (DAS). As such, an increase in the length of cloudlets signifies an increase in the length of the fiber used for DAS sensing. The data presented in Figure 11 indicate a clear trend: as the length of the cloudlets increases, so does the processing time. This trend is consistent across both single-cycle and multi-cycle measurements. The underlying reason for this observed trend is the increased number of differential computations required as the length of the fiber increases. In essence, the longer the fiber used for DAS sensing, the more data points are generated, and consequently, the more differential computations are required. This increase in computational demand results in a corresponding increase in processing time. Each VM is represented by a line of a different color. The arrangement from top to bottom indicates their respective positions on the graph, with VM 1 at the top and VM 10 at the bottom.

The graph shows almost a linear relationship between the Cloudlet ID and the processing time in seconds for each VM. This suggests that as the number of cloudlets increases, the processing time for each VM also increases at a consistent rate. VM 1, represented by the green line, has the steepest slope among all the VMs. This indicates that its processing time increases most rapidly with the number of cloudlets, which implies that VM 1 is the least efficient or is handling more complex tasks that require more processing time. On the other hand, VMs 2 to 10, represented by lines of different colors, have less steep slopes compared to VM 1. This shows that their processing times increase more gradually with the number of cloudlets. The top-to-bottom arrangement of the lines provides a clear comparison of efficiency across the VMs. VM 1 has the highest processing times, while VM 10 has the lowest. This shows that VM 10 is the most efficient in terms of processing time. This graph provides valuable insights into the performance of each VM, allowing for an assessment of their efficiency and capacity for processing cloudlets during the differential operation. As we can see from the figures, as the length of the fiber increases, the increase rate in the processing time decreases more and more. We will discuss this further.

**Figure 12:**
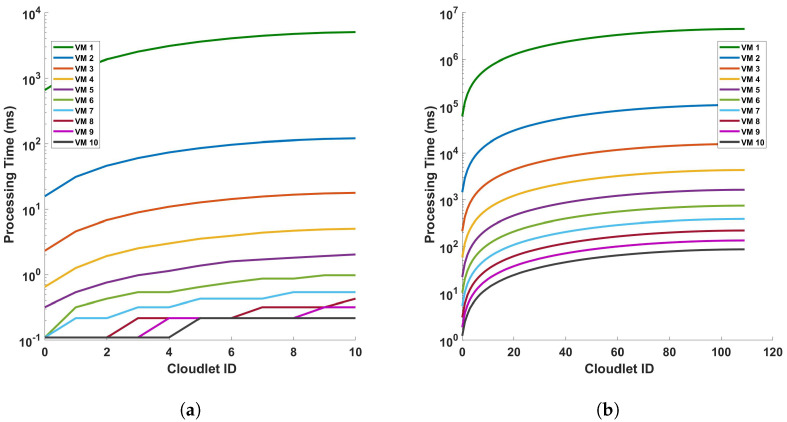
Processing time versus cloudlets for FFT operation for (**a**) a single cycle, and (**b**) 10 cycles, of measurement in a 110 km fiber. Note that the number of cloudlets increases for each cloudlet ID in the horizontal axis.

Figure 12 illustrates the relationship between the processing time and cloudlet length for Fast Fourier Transform (FFT) operations. This relationship is examined under two distinct scenarios, a single cycle of measurement and ten cycles of measurement, both conducted within a 110 km fiber optic cable. As such, an increase in the length of the cloudlets signifies an increase in the length of the fiber used for DAS sensing. The data presented in Figure 12 indicate a clear trend: as the length of the cloudlets increases, so does the processing time. This trend is consistent across both single-cycle and multi-cycle measurements. The underlying reason for this observed trend is the increased number of FFT computations required as the length of the fiber increases. In essence, the longer the fiber used for DAS sensing, the more data points are generated, and consequently, the more FFT computations are required. This increase in computational demand results in a corresponding increase in processing time. This observation aligns with the fundamental principles of computational science, providing a reasonable explanation for the trend depicted in Figure 12. It underscores the computational implications of using longer fibers in DAS sensing and highlights the need for efficient computational strategies to manage the increased processing demand. The explanation that were given for the differential operation can be given here. Comparing this FFT operation graph to the previous one with subtraction operation, we can infer that the FFT operation adds computational complexity, resulting in increased processing times for the VMs, which is also clear from the steepness of the lines in the FFT graph compared to the differential graph. This can provide insights into how much additional processing time the FFT operation requires for each VM. This analysis can help in understanding the impact of FFT operations on VM performance and in making decisions about resource allocation and optimization for computational tasks involving FFTs.

### 3.3. Evaluation of Different Statistical Characteristics of Signal Processing in a DAS Using CloudSim

Various statistical characteristics like mean, standard deviation, and variance of the signal processing will be evaluated in this section.

**Figure 13:**
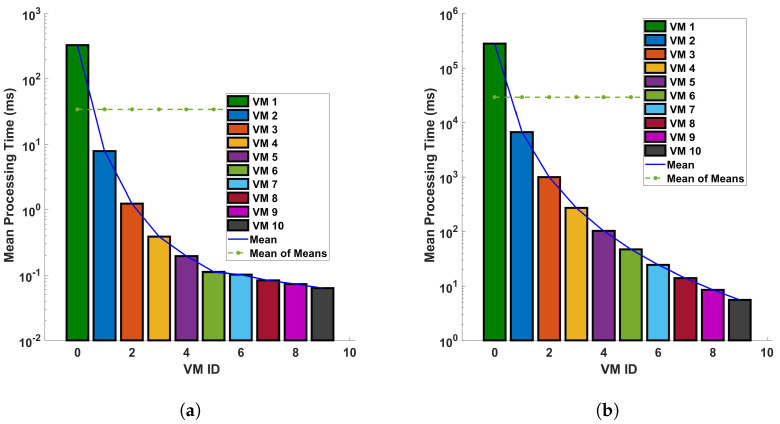
Evaluation of the mean processing time for each virtual machine in differential operations: a comparative study on a single cycle versus multiple cycles in a 110 km optical fiber. The investigation is conducted under two distinct conditions: (**a**) a single cycle of measurement, and (**b**) a series of 10 consecutive cycles of measurement. The measurements are performed in a 110 km long optical fiber. This research aims to understand the computational efficiency of cloud services in DAS sensing systems.

**Figure 14:**
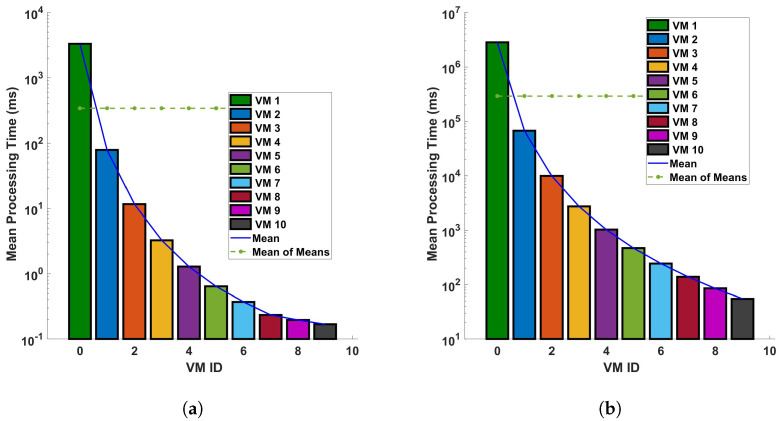
Mean processing time for each VM for FFT operation for (**a**) a single cycle, and (**b**) 10 cycles, of measurement in a 110 km fiber.

Figure 13 and Figure 14 provide a comparative analysis of the mean processing time for two distinct operations—differential and Fast Fourier Transform (FFT)—under two different conditions: (a) a single cycle of measurement, and (b) ten cycles of measurement. These measurements are conducted within a fiber optic cable that spans a distance of 110 km. The figures indicate a clear trend: the processing time decreases significantly as we transition to a more capable virtual machine (VM). The underlying reason for this observed trend is the efficiency of the more capable VM in executing differential and FFT computations. Simply put, a more powerful VM can process larger datasets—in this case, data from the fiber optic cable—in a shorter amount of time. This results in a significant reduction in processing time as depicted in Figure 13 and Figure 14. These figures underscore the importance of computational power in processing large datasets, particularly in the context of differential and FFT operations in Distributed Acoustic Sensing (DAS). They highlight the efficiency gains that can be achieved by utilizing more capable VMs, thereby emphasizing the need for strategic resource allocation in computational tasks. In all plots, there is a trend of decreasing processing time with increasing VM ID. This indicates that VMs with higher IDs are more efficient or have been optimized for the different computations like the differential and FFT. The difference in precise measurements (for a single cycle and 10 cycles of measurement) affects the processing time. We can see that when the precision of measurements is increased, the mean of the computation time for differential operation also increases. As the precision is increased more, the trend of the decreasing processing time with increasing VM ID decreases; this indicates that the performance characteristics of the VMs are stable regardless of the operation distance and the type of operation. Note that a typical application of the cloud computing system is pipeline safety and integrity monitoring, which requires long-range sensing for leakage detection. The processing time for a 110 km fiber has typical values of up to a few ms for some virtual machine configurations, which means it could enable the monitoring of vibrations with frequencies of a few 100 s of Hz in real time. In addition, we have considered 5 sampling points for each meter of fiber, and the benchmark processing scheme is suitable for monitoring with sub-meter spatial resolution.

**Figure 15:**
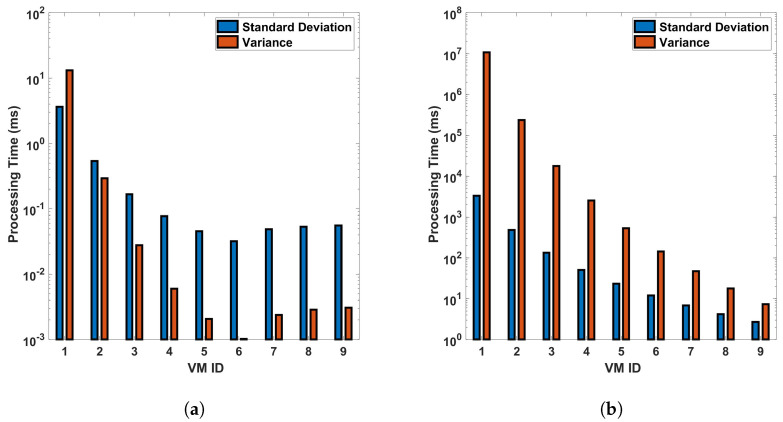
Statistical analysis of processing time for virtual machines in differential operations: an examination of standard deviation and variance across single and multiple cycles in a 110 km optical fiber. The analysis is conducted under two different scenarios: (**a**) a single cycle of measurement, and (**b**) a sequence of 10 cycles of measurement. The measurements are carried out in a 110 km long optical fiber. This study provides a deeper understanding of the variability and consistency in the performance of VMs during differential operations in DAS sensing systems.

**Figure 16:**
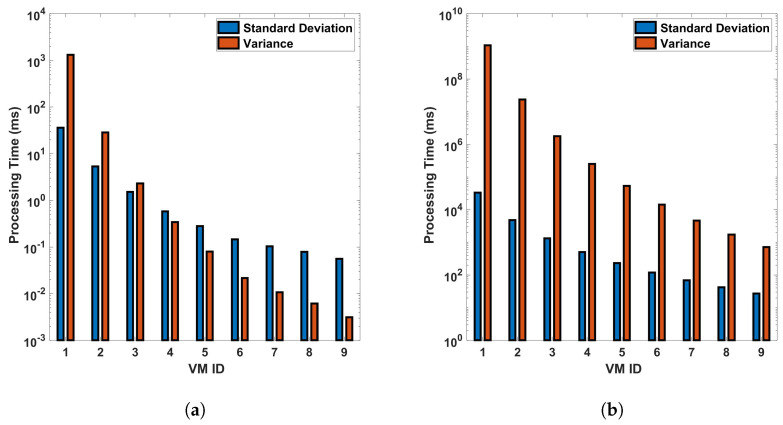
Standard deviation and variance for vms based on processing time-for differential operation for (**a**) a single cycle, and (**b**) 10 cycles, of measurement in a 110 km fiber.

From Figure 15 and Figure 16, we can see that VM ID 1 has the highest standard deviation and variance among all, with both values approximately 1500 ms in the differential process for a single cycle of measurement in a 110 km fiber. These values become even larger for the FFT operation, larger distances, and more precise measurements. This shows that VM 1 has the most variability in processing time. VM IDs 2, 3, 4, and 5 show a similar pattern, where the standard deviation is higher than the variance but lower than that of VM ID 1. This indicates that these VMs also have a significant amount of variability in processing time but less than VM 1. On the other hand, VM IDs 6 through 10 show a decrease in both the standard deviation and variance, with VM ID 8 having the lowest values. This suggests that these VMs have the least variability in processing time. In general, a higher standard deviation or variance indicates more variability or dispersion in the data. In this context, VMs with lower values (VM 1, VM 2...) have more inconsistent processing times, which could be due to various factors such as workload, hardware performance, etc. Conversely, VMs with higher values (VM 10, VM 9, etc.) have more consistent processing times. Note that standard deviation measures how far apart from each other our values are in a dataset. As we can see from the bar plots, which show a decrement from VM 0 to VM 9, it is suggested that the variance in processing time decreases across the VMs. This means that the earlier VMs have more variability in their processing times, while the later ones are more consistent, as we have seen earlier from previous plots. The line representing the mean also decreases, indicating that the average processing time is reducing as we move from VM 0 to VM 9. This trend implies that the later VMs are not only more consistent but also faster on average than the earlier ones. This analysis shows the relationship between the variance and the mean processing time and indicates that the high configuration of VMs contributes to both the consistency and efficiency of the VMs as their IDs increase (as the parameters of the VMs increase). The VMs with smaller standard deviation lines are more reliable in terms of consistent performance, whereas VMs with larger standard deviation lines have more fluctuation in their processing times.

### 3.4. Evaluation of Scalability of Signal Processing in a DAS Using CloudSim

One important aspect when dealing with a system is that it is scalable as input data become bigger and bigger. In this section, we will assess the scalability of our cloud computing system, particularly focusing on its ability to handle increasing input data sizes effectively.

**Figure 17:**
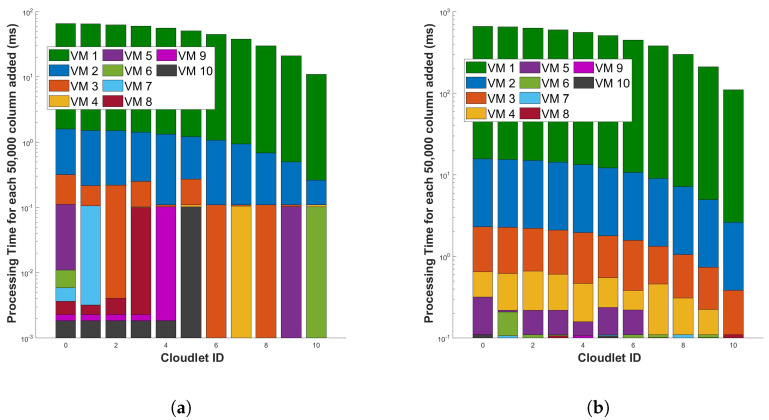
Evaluation of processing time for incremental data in optical fiber measurements (for each additional 50,000 rows) during two distinct operations: (**a**) the differential operation, and (**b**) the Fast Fourier Transform (FFT) operation. The measurements are conducted in a 110 km long optical fiber. This examination aims to understand the computational scalability of these operations in the context of increasing data volume.

Figure 17 represents the processing time for differential and FFT operation for a single cycle of measurement in a 110 km fiber, respectively. The figures show that the processing time required for every new 50,000 columns added (when the length of the fiber is increased) decreases significantly as the length of the fiber used for the sensor increases. This is because the more capable VM will execute differential and FFT computations in parallel and will utilize the VM fully. In the graph, as the number of batch of columns added to the cloudlets increases, we observe that the processing time for each individual batch of columns added to the cloudlet is decreasing. This is because, with more data, the computational resources such as CPUs or GPUs can be utilized more efficiently. The parallel processing capabilities of these resources mean that they can handle larger batches of data more effectively than smaller ones. Additionally, the overhead costs associated with setting up each batch for processing are distributed across a larger batch of columns added to the cloudlets. So, while the total processing time for all cloudlets might increase with more data, the time attributed to each individual batch of columns added to the cloudlet (or the average processing time per batch of columns added to the cloudlet) is decreasing, which shows that our cloud computing system is scalable. However, it is important to note that this trend will only hold up to a certain point. If the batch size becomes too large, it may exceed the memory capacity of the hardware, or the efficiency gains from parallel processing may plateau, leading to no further reductions in processing time per cloudlet.

### 3.5. Evaluation of the Effect of Different Parameters of VM on Computation Times of Signal Processing in a DAS Using CloudSim

In this section, we will investigate whether the different parameters of VM like the MIPS and PE will affect the performance of our system separately.

**Figure 18:**
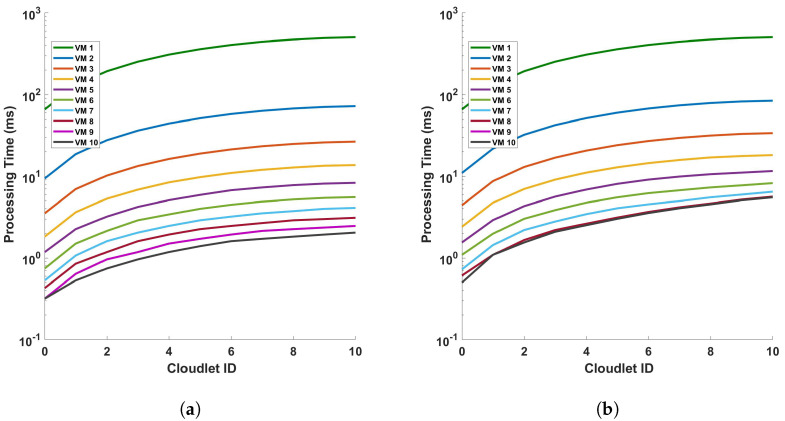
Analysis of processing time and cloudlet utilization for differential operations in optical fiber measurements with a specific focus on two distinct scenarios: (**a**) varying only the Million Instructions Per Second (MIPS) of the virtual machines (VMs), and (**b**) varying only the Processing Elements (PE) of the VMs. The measurements are conducted during a single cycle in a 110 km long optical fiber. This study aims to understand the influence of MIPS and PE variations on the performance and efficiency of VMs during differential operations in DAS sensing systems.

Figure 18 provides a detailed analysis of the processing time in relation to the length of cloudlets for differential operations under two distinct scenarios. Both scenarios involve a single cycle of measurement within a 110 km fiber optic cable, but they differ in the parameters of the virtual machines (VMs) that are varied: (a) in the first scenario, the processing capability of the VMs, represented by the Million Instructions Per Second (MIPS), is varied while all other parameters remain constant, and (b) in the second scenario, the number of Processing Elements (PEs) of the VMs is varied, again, with all other parameters held constant. The figures indicate a clear trend: both the MIPS and PE parameters of the VMs have a direct impact on the performance of the computation. They highlight the efficiency gains that can be achieved by optimizing the MIPS and PE parameters of the VMs, thereby emphasizing the need for strategic resource allocation in computational tasks. Figure 18 offers valuable insights into the relationship between the processing time, the length of the cloudlets, and the computational capabilities of the VMs used for DAS sensing. It underscores the computational implications of using VMs with different MIPS and PE parameters and highlights the need for efficient computational strategies to manage the increased processing demand. It is the MIPS and PE that directly affect the computation. The processing time for VM 1 is the same when we vary the different parameters because VM1 have always the same parameters. Hence, the effect is only seen on the other VMs.

**Figure 19:**
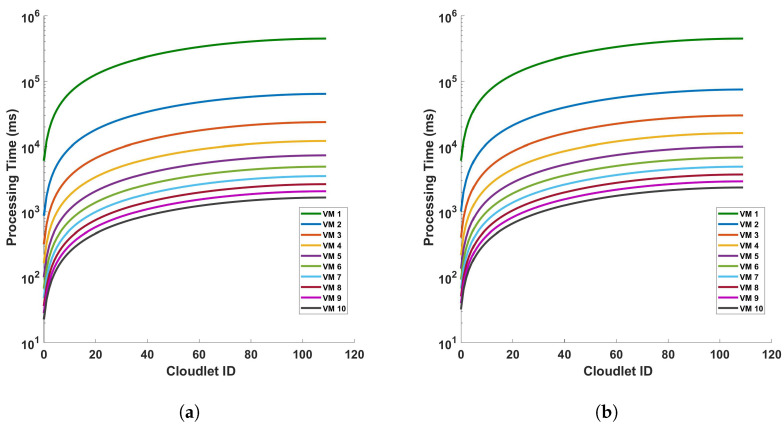
Processing time versus cloudlets for differential operation for (**a**) varying only the MIPS of the VMs, and (**b**) varying only the PE of the VMs, for a 10 cycle of measurements in a 110 km fiber.

Figure 19 shows the (a) processing time versus cloudlets for differential operation for a 10 cycle of measurements in a 110 km fiber while varying only the MIPS of the VMs, and (b) the processing time versus cloudlets for a differential operation for 10 cycles of measurements in a 110 km fiber while varying only the PE of the VMs. The same analysis in that of Figure 18 applies here too.

### 3.6. Cost Analysis for Computation Time and Computation Distance of Fiber of Signal Processing in a DAS Using CloudSim

In this section, we will analyze the computational time costs for signal processing and the computation costs associated with the fiber length.

**Figure 20:**
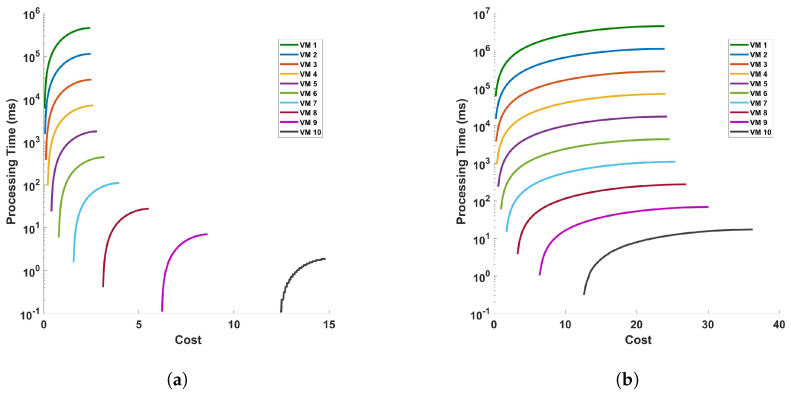
Processing time versus cost for (**a**) differential, and (**b**) FFT operation for 10 cycles of measurement in a 110 km fiber.

Figure 20 represents the processing time versus cost for (a) differential, and (b) FFT operation for 10 cycles of measurement in a 110 km fiber. The figures show that cost increases as the processing time increase. This is because the differential and FFT computations are performed for longer time in the VM. The plot indicates that as the cost associated with a VM increases, the processing time for the operation generally decreases. This trend shows that more expensive VMs have better performance, likely due to higher specifications or better resources, which allow them to process tasks more quickly. However, there is an exception noted by a green line on the plot (VM 1), where this particular VM exhibits a sharp increase in processing time despite an increase in cost. This anomaly could be due to various factors such as inefficient resource allocation, sub-optimal configuration, or other issues because we are using VM with very low configurations for complex and large computations. So, we should avoid using this type of VM when dealing with complex and large computations.

**Figure 21:**
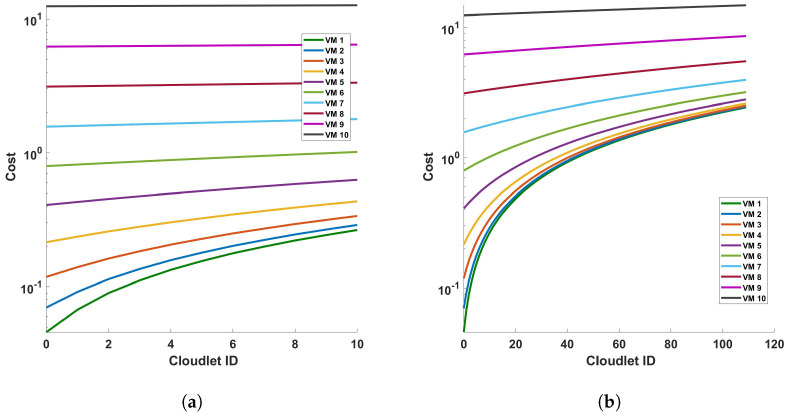
Cost of processing versus cloudlets for differential operation for (**a**) a single cycle, and (**b**) 10 cycles, of measurement in a 110 km fiber.

**Figure 22:**
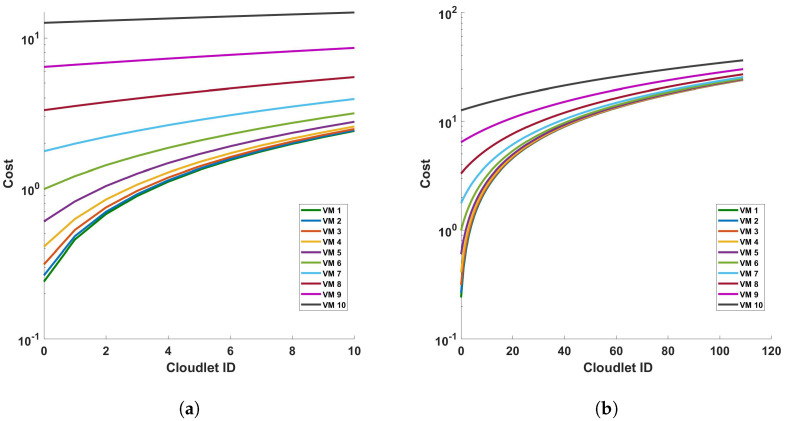
Cost of processing versus cloudlets for FFT operation for (**a**) a single cycle, and (**b**) 10 cycles, of measurement in a 110 km fiber.

Figure 21 and Figure 22 represent the cost of processing time versus cloulets for differential and FFT operation for (a) a single cycle, and (b) 10 cycles, of measurement in a 110 km fiber. The figures show that the cost increases as the length of the cloudlets increases or the accuracy of the measurement is increased. This is because the differential and FFT computations are performed for more columns (for more length of the fiber and/or for accurate measurements). On both figures, we have figures for (a) a single cycle, and (b) 10 cycles, of measurement in a 110 km fiber to produce more precise plots. Comparing the figures, we see that both operations exhibit a trend where the processing time increases with the cloudlet ID. Since the cloudlet length represents the computations for the the length of the fiber, we can see that the processing time increases as the length of the fiber increases and as the computation becomes complex. FFT operations are computationally intensive, so we expect them to have longer processing times compared to differential operations; this is also explicitly indicated by the plots. Specifically, we can also see from the plot (a) of the Figure 21 that the increase in the cost from cloudlet0 to cloudlet10 is ∼$0.2, i.e., as we change the length of the fiber from 1 km to 110 km, and also when we change the VMs choice, the cost increases between consecutive VMs.

## 4. Conclusions

In summary, we have demonstrated the capacity of the Amazon DynamoDB service to be used in scalable storage in a DAS based on a phase-sensitive OTDR, using as the benchmark a 100 km sensing distance with a meter-scale spatial resolution. The latency per batch of data access shows a consistent mean value of ∼40 ms with low standard deviation values for both linearly and nonlinearly varying numbers of batches of data. In addition, the latency per sample for sampling points corresponding to the sensing distances of 1–110 km converges to an optimum value as the number of samples increases. Our study suggests a strong potential for DynamoDB to address one of the open issues of efficient and scalable data storage and handling in not only DAS but also many distributed optical fiber sensors, and could lead to the wider development of scalable data handling schemes, including those leveraging signal processing with cloud computing services.

In addition, in the second part of our work, we have shown that the cloud computing is capable of supporting different computations that are needed for the extraction of sensing data from the large DAS sensor data. To show this, we will use a potent computational system using CloudSim for managing sensor data in the cloud. The system comprises a robust data center, agile cloudlets, and virtual machines (VMs) with diverse configurations, processing power, memory, storage, and networking capabilities. Through simulations focused on batch-processing tasks like differential and Fast Fourier Transform (FFT) operations, the system demonstrates its prowess in efficient data handling. These simulations yield valuable insights into the key performance metrics necessary for signal processing for dynamic monitoring with a DAS as well as resource optimization strategies and cost-effectiveness analysis showcasing the system’s effectiveness in cloud-based computational environments.

The analysis delves into the relationship between the processing time, cloudlets, cost, and various virtual machine (VM) parameters in the context of differential and Fast Fourier Transform (FFT) operations across different fiber lengths in a Distributed Acoustic Sensing (DAS) system using CloudSim simulations. Computations of the processing time versus cloudlets for both operations for a single cycle and 10 cycles of measurement in a 110 km fiber demonstrate a clear trend of increasing processing time with longer cloudlets, reflecting the greater number of computations required as the fiber length (and thus, the columns processed) increases. It also confirms the scalability of the signal-processing system for larger numbers of cycles of measurements, hence demonstrating that the design scheme is suitable for real-time dynamic monitoring.

Moreover, analyses of the VM performance in terms of the the mean processing time across different VMs show a significant decrease in the processing time with more capable VMs. This trend confirms the importance of VM selection in optimizing system performance and efficiency. Additionally, the examination of standard deviation among VMs reveals insights into processing time variability, with the VM having the lowest performance (processing speed, number of CPUs) exhibiting the highest variability and higher-performance VMs showing decreasing variability and lower average processing times. Further investigation into the impact of VM parameters elucidates that an increase in the Processing Elements (PE) and Million Instructions Per Second (MIPS) reduces the processing time. Additionally, the analysis observes performance trends with increasing fiber length, demonstrating a notable decrease in the processing time as the fiber length (and cloudlets) increases. The observed decrease in processing time for each new set of 50,000 columns added as the fiber length increases highlights the system’s scalability and efficiency in handling larger computational workloads. This efficiency gain, coupled with the correlation between processing time, cost, and VM parameters, underscores the importance of resource allocation and configuration optimization for achieving optimal performance and cost effectiveness in DAS systems. These findings contribute to a deeper understanding of system dynamics and inform decision-making processes in designing and managing processing resources in DAS for real-time monitoring in diverse applications. This trend suggests improved system performance and efficiency with larger fiber lengths, reflecting the capabilities of more extensive computational resources.

Moving to the cost analysis, it reveals a direct relation between the processing time and cost, as longer processing times in VMs for both differential and FFT computations lead to higher costs. Similarly, examining cost versus cloudlets for differential and FFT operations shows an upward trend in cost with longer cloudlets, indicating increased processing requirements for larger fiber lengths. But the cost does not increase significantly as the fiber length and or the VM parameters are changed. These findings highlight that longer fiber lengths can be used for sensing, while not incurring significantly higher prices.

In summary, we can see that the analysis provides valuable insights into the interplay between the processing time, VM parameters, cost, and system performance in DAS systems, offering a nuanced understanding of resource utilization, optimization strategies, cost, and performance trends across varying computational requirements, fiber lengths, and measurement accuracies.

## Data Availability

Data are contained within the article.

## Abbreviations

The following abbreviations are used in this manuscript:

AWS	Amazon Web Services
DAS	Distributed Acoustic Sensing
DB	Data base
FBG	Fiber Bragg Grating
FFT	Fast Fourier Transform
IaaS	Cloud Infrastructure as a service
IOT	Internet of Things
MI	Million Instruction
MIPS	Million Instruction Per Second
NoSQL	Not only SQL
OTDR	Optical Time Domain Reflectometer
PaaS	Cloud Platform as service
PE	Processing Elements
RBS	Rayleigh back-scattering
RTT	Round-Trip Time
SaaS	Cloud software as a service
SNR	Signal-to-Noise Ratio
SQL	Sequential Query Language
SWI	Swept Wavelength Interferometry
VM	Virtual Machine
